# Modulation of Enterohaemorrhagic *Escherichia coli* Survival and Virulence in the Human Gastrointestinal Tract

**DOI:** 10.3390/microorganisms6040115

**Published:** 2018-11-19

**Authors:** Grégory Jubelin, Mickaël Desvaux, Stephanie Schüller, Lucie Etienne-Mesmin, Maite Muniesa, Stéphanie Blanquet-Diot

**Affiliations:** 1 UMR454 MEDIS, Université Clermont Auvergne/INRA, 63000 Clermont-Ferrand, France; gregory.jubelin@inra.fr (G.J.); mickael.desvaux@inra.fr (M.D.); lucie.etienne-mesmin@uca.fr (L.E.-M.); 2 Norwich Medical School, University of East Anglia, Norwich NR4 7TJ, UK; s.schuller@uea.ac.uk; 3 Gut Health and Food Safety Programme, Quadram Institute Bioscience, Norwich NR4 7UA, UK; 4 Department of Genetics, Microbiology and Statistics, Microbiology Section, Faculty of Biology, University of Barcelona, 08029 Barcelona, Spain; mmuniesa@ub.edu

**Keywords:** EHEC, virulence factors, in vitro GI models

## Abstract

Enterohaemorrhagic *Escherichia coli* (EHEC) is a major foodborne pathogen responsible for human diseases ranging from diarrhoea to life-threatening complications. Survival of the pathogen and modulation of virulence gene expression along the human gastrointestinal tract (GIT) are key features in bacterial pathogenesis, but remain poorly described, due to a paucity of relevant model systems. This review will provide an overview of the in vitro and in vivo studies investigating the effect of abiotic (e.g., gastric acid, bile, low oxygen concentration or fluid shear) and biotic (e.g., gut microbiota, short chain fatty acids or host hormones) parameters of the human gut on EHEC survival and/or virulence (especially in relation with motility, adhesion and toxin production). Despite their relevance, these studies display important limitations considering the complexity of the human digestive environment. These include the evaluation of only one single digestive parameter at a time, lack of dynamic flux and compartmentalization, and the absence of a complex human gut microbiota. In a last part of the review, we will discuss how dynamic multi-compartmental in vitro models of the human gut represent a novel platform for elucidating spatial and temporal modulation of EHEC survival and virulence along the GIT, and provide new insights into EHEC pathogenesis.

## 1. Introduction

Enterohaemorrhagic *Escherichia coli* (EHEC) is an important cause of human gastrointestinal (GI) disease in developed countries [1]. Ruminants, especially cattle, are a natural reservoir of the pathogen, and food such as undercooked beef products, unpasteurized milk, and vegetables, as well as contaminated water, are the principal sources of human infection. EHEC causes diarrhoea, haemorrhagic colitis, and systemic haemolytic uraemic syndrome (HUS) in 10% of cases. HUS is the leading cause of acute renal failure in children, and up to 5% of patients die from HUS [2]. The severity of HUS combined with the very low infectious dose (around 50–100 bacteria) makes EHEC a very serious pathogen. EHEC contains over 400 serotypes, but O157:H7 is most often associated with outbreaks worldwide and severe diseases [3].

Survival and virulence of EHEC strains in the human gastrointestinal tract (GIT) are key factors in the infectious process. Once ingested, the pathogen must breach the acidic barrier of the human stomach to reach its main colonization site, the terminal ileum and colon [4,5,6]. EHEC colonization involves the formation of attaching and effacing (A/E) lesions on intestinal epithelium, which are characterized by loss of microvilli and intimate attachment to the host cell membrane [7]. Genes encoding A/E lesion formation are localized on a pathogenicity island, the locus for enterocyte effacement (LEE), which encodes a bacterial type III secretion system (T3SS). Colonization is mainly mediated by the primary adhesin intimin but other putative adherence factors have been described, such as long polar fimbriae (Lpf) [8]. In addition, mucin-degrading enzymes such as the secreted zinc metalloprotease StcE promote penetration of the mucus layer and bacterial adhesion [9]. After establishment of colonization, Shiga toxins (Stx), the main virulence factors of EHEC responsible for HUS, are released and cross the epithelial barrier to reach their target organs, the kidneys and the brain [10]. Stx are encoded in the genomes of lambdoid bacteriophages and consist of five B subunits binding to globotriaosylceramide-3(Gb3) receptors on the surface of endothelial target cells and one catalytic A subunit targeting eukaryotic ribosomes and inhibiting protein synthesis [11]. Stx production is directly dependent on phage induction, which is induced by activation of the bacterial SOS response by DNA damaging agents, such as antibiotics [12]. The Stx family has two main groups: Stx1 and Stx2. Stx2 is only produced when the phage enters the lytic cycle, while Stx1 is regulated by phage cycle and an iron-regulated promoter [13]. Hence, Fur, a ferric uptake regulator protein, represses Stx1 gene expression when high levels of iron are present. This illustrates that EHEC virulence is not dependent on a single gene or gene product but is a multifactorial process.

**Figure 1:**
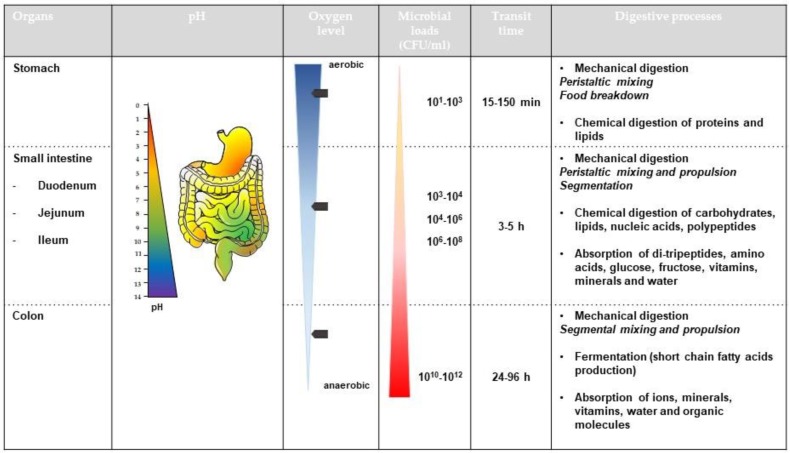
Overview of the main physical and chemical processes of the human gastro-intestinal tract.

Despite its key role in bacterial pathogenesis, the modulation of EHEC survival and expression of virulence genes along the human GIT remains largely unknown as studies of human volunteers are unethical [14]. This review aims to give an overview of in vitro (simple models of the human gut) and in vivo (animal models) studies describing the effect of GI cues on EHEC viability and virulence including motility, adhesion, phage induction and Stx production. Considering the complexity of human digestive physiology (Figure 1), this review will address the influence of numerous abiotic (e.g., physicochemical parameters such as pH, bile, low oxygen concentration or digestive enzymes) and biotic (e.g., intestinal microbiota and host mucosa) factors of the human gut on EHEC infection. Lastly, we will discuss how well-controlled and validated dynamic and multi-parametric in vitro models of the human gut can help address knowledge gaps in EHEC pathogenesis.

This figure summarizes the key physico-chemical and microbial processes occurring in the gastrointestinal tract of healthy human adults

## 2. Effect of Abiotic Parameters of the Human Gut

### 2.1. Effect of pH

#### 2.1.1. Variations in pH along the Human Gut

The pH of the human GIT is extremely variable. In the mouth, the pH of saliva is usually between 6.5 and 7.5 and can be altered by the pH of masticated food. After swallowing, the food enters the upper part of the stomach, where the pH decreases to 4–6.5. Digestion occurs, mainly in the lower part of the stomach, by secretion of hydrochloric acid (HCI) and pepsin, which further reduces the pH from 4 to an extremely acid pH of around 1.5. In the small intestine, the pH rises to 6 in the duodenum and reaches about 7.4 in the terminal ileum. While the pH drops to 5.7 in the caecum, it gradually increases again to a value of 6.7–7 in the distal colon [15].

#### 2.1.2. Effect of pH on EHEC Survival and Physiological State

Regarded as a neutrophile, EHEC, like other *E. coli*, grows well in conditions of near neutrality, at pH values of 6 to 8. It also grows, although more slowly, at pH values of 5 and 9. However, regardless of the value of the pH outside the bacteria, the internal pH (pHi) is maintained close to 7.6 [16]. Many enzymes necessary for microbial growth only work within this narrow pH range. This suggests that in the human gut, EHEC has to adapt to the wide range of GI pH (especially acidic pH of the stomach) to maintain pHi homeostasis. This is accomplished by the buffer capacity of the bacterial cell, where the outward transport of protons associated with respiration and ATP hydrolysis creates a transmembrane gradient [17], and electroneutral antiport systems exchange protons for certain cations, particularly Na^+^ and K^+^ [17].

The resistance of EHEC to low pH conditions has been widely reported [18,19,20] and even if there are strain-specific variations with regard to acid tolerance, EHEC have a greater ability to survive in complex acidic environments than commensal *E. coli* [21]. This ability to resist an acidic environment contributes to its low infectious dose by allowing small numbers of the organism to pass through the stomach acidity barrier [20]. When the growth capacities of different EHEC O157:H7 strains were compared before and after exposure to acidic stress, differences in viability of no greater than a log-fold were observed, indicating that they were able to withstand both brief acute acid stress and acid-adapted acute acid stress [22,23]. These results are supported by an evaluation of the wild type strain EDL933 over a broad pH range (3 to 10). While the strain showed an optimal growth at pH 5.5–8.5 [24], this was only reduced by a log-fold at pH 4, and growth of culturable cells was even observed at pH 3 and at 10, although to a lesser extent. 

*E. coli* O157:H7 adapted to pHs of 5–9 exhibited changes in their membrane lipid composition, which in turn affected membrane fluidity, with more evident alterations observed in acidic conditions [23]. The decrease in membrane fluidity under acidic growth conditions may be associated with changes in proton flux, whereby adapted cells do not allow protons to flow into the cell as easily as non-adapted cells [23]. The decrease in membrane fluidity may increase acid resistance. Acid adaptation also reduced the total amount of verotoxin produced, perhaps by repressing the production of toxin [23].

Besides the mechanisms of adaptation, *E. coli* displays a complex set of stress response mechanisms that bestow an ability to survive, or even thrive, in acidic conditions. Components of the growth medium, particularly arginine, lysine, ornithine and glutamic acid, were reported to enhance the acid resistance of fermentative cells [25,26]. Acid resistance in enteric bacteria such as EHEC involves enzyme-based responses to acid stress classified in five acid resistance (AR) pathways. AR1 is an oxidative system and its mechanism of action is the least known [27]. AR2, AR3, AR4 and AR5 are glutamate (GAD system)-, arginine-, lysine- and ornithine-decarboxylase pathways, respectively. Each of the amino acid decarboxylase-based pathways (AR2–AR5) is activated under different conditions and is based on decarboxylation reactions that consume a proton and release carbon dioxide in return [26]. These reactions are complemented by chaperon-based acid resistance systems, in which HdeA and HdeB protect periplasmic proteins, which are more vulnerable to acid denaturation and damage [28]. Gene expression assays show clear up-regulation of numerous decarboxylase genes after prolonged acid stress [22], confirming the involvement of the amino-acid decarboxylase-based AR pathways after acid stress [26]. Moreover, the presence of the Stx phage-encoded transcriptional regulator CII was identified as the controller of the acid response in Stx lysogens and, by upregulation of the GAD operon (AR2 pathway), an Stx2 phage enhanced acid resistance in Stx lysogens [29].

#### 2.1.3. Effect of pH on EHEC Virulence

The pH in general and acid stress in particular, affects several critical virulence properties of EHEC, including increase in motility and host adhesion, iron uptake, and repression of Stx phage induction (with consequently no increase of Stx production). Moreover, epithelial cells infected with acid-stress EHEC O157:H7 showed significantly higher levels of apoptosis than cells infected with unstressed bacteria [22].

##### Motility

In the non-pathogenic strain *E. coli* K-12, flagellar and chemotaxis genes are repressed at pH 8.7, likely by inhibition of the proton pumps and proton retention. This results in reduced motility, as protons are responsible for flagella synthesis and motive force [30]. In contrast, low pH accelerates acid consumption and proton export, thereby enhancing motility [30]. In EHEC O157:H7, motility was slightly increased in acute acid-stress conditions compared with no acid stress [22]. Protein H-NS, a nucleoid-associated protein, seems to play a role in the regulation of bacterial motility in response to low pH [30]. Interestingly, some results indicate that motility is affected more by the variation between extracellular pH (pHo) and pHi than by the external pH. It has been reported that if the pHo is acidic, the motility of *E. coli* is impaired, suggesting that the loss of motility could be caused by the change in cytoplasmic pH [31]. The subsequent increase in the intracellular proton concentration interferes with the release of protons from the torque-generating units, resulting in a slowing or stopping of the flagellum motors [32].

##### Adhesion

Compared to unstressed bacteria, acid-stressed EHEC O157:H7 adhered more effectively to different cell lines [22], even after subtle pH changes. In contrast, commensal *E. coli* did not show significant differences in adhesion regardless of the acidic conditions [22]. Gene array studies found that prolonged acid stress caused a significant up-regulation in the expression of flagella genes, T3SS proteins, adhesion and fimbrial proteins, but not housekeeping genes [22]. *Lpf 2* expression was also four-fold higher at pH 6.5 than at pH 7.2, which might be important for adhesion and colonization of the small intestine [33].

##### Iron Uptake

In addition to iron-utilizing systems, Fur and iron regulate a variety of genes involved in *E. coli* acid resistance, thereby linking the uptake and availability of iron to the response to variations in pH [34].

##### Stx Phage Induction and Stx Production

The induction of Stx phages was inhibited at pH 3 and 4, both in control cultures and those treated with inducing agents like mitomycin C [24]. The inhibition of Stx2 phage induction at a low pH indicates the presence of some other, yet unknown, regulator that prevents phage induction. Consequently, lysogen survival after the lysis caused by phage induction is higher in acidic environments, and this protective effect can be considered as an important virulence factor. The number of Stx phages induced from EHEC increased at pH 5.5–8.5, reaching a maximum at pH 7, while their number decreased at pH 10 [24]. Little and colleagues also reported that the purified lambda phage repressor underwent self-cleavage at alkaline pH in vitro [35]. Other studies showed activation of the SOS system by alkaline pHi in *E. coli* for Stx phage induction [36]. This means that alkaline pH could lead to phage spontaneous induction [37], that causes increase of *stx* expression when pH increases and in the absence of an inducing agent. Preferential Stx phage induction at neutral and alkaline conditions might be explained by the need to release and activate Stx in the intestine, where pH values are higher.

Low pH also inhibits phage infection [38] and consequently the possible transduction of *stx* by Stx phages [39]. In fact, lysogenic conversion by Stx phages seems almost impossible in a low pH environment, possibly because phages cannot attach to the host cell at a pH of 3, which affects proteins responsible for phage attachment. The phage capsid apparently provides the DNA with enough protection in almost all conditions, except perhaps those of a low pH. The reported instability of the bacteriophage capsid at low pH values (<4) [40,41] could explain why results obtained at pH 3 differed from those at pH 7 or 9. A low pH, below or equal to the phage isolectric point, has also been shown to cause phage aggregation [42].

A direct consequence of the repression of Stx phage induction, particularly evident at low pH, is the lack of expression of *stx* even under phage-inducing conditions. In accordance to the study by Imamovic and Muniesa [24], the production of Stx by *E. coli* O157:H7 in syncase broth was optimal at a pH of 8 to 8.5 [24]. Some studies attribute the inhibition of Stx production by probiotic bifidobacteria [43] partly to a low pH. The adaptation of cells to pH conditions also influenced Stx production, which was lower in acid-adapted than non-adapted and alkali-adapted bacteria [23,44]. Moreover, acid-adapted EHEC at pH 5.6 produced less Stx than non-acid-adapted bacteria at pH 7.4 after 48 hours. Other authors do not report any significant changes of Stx production at low pH [22] and this may seem contradictory. In their study, the authors used an experimental approach where Stx production was not assayed under Stx phage inducing conditions, which is when the reduced *stx* expression as a consequence of the repressed phage induction becomes more evident. Consequently, the microarray results show no changes of *stx* expression compared with the basal *stx* expression produced at neutral pH, also under non-inducing conditions.

All together, these data suggest that EHEC strains orchestrate a complex machinery including expression of virulence determinants in response to acidic environment of the stomach.

### 2.2. Effect of Bile Salts

#### 2.2.1. Bile Salts in the Gut

The emulsification and solubilisation of dietary lipids and elimination of substances that cannot be efficiently excreted in urine is accomplished by bile. Bile is a complex digestive secretion produced by the liver (one liter per day), and is composed of a high amount of lipids and a low amount of proteins. The major constituents of bile are bile acids, which are present in the small intestine in a conjugated form (bile salts) at a concentration that generally varies from 0.2 to 2.0%, although dietary intake and nourishment status can greatly affect bile levels. The concentration decreases from 10 mM in the duodenum to 2 mM in the ileum as bile salts are passively reabsorbed along the entire length of the small intestine and then actively reabsorbed in the ileum [45]. The concentration gradient in the small intestine and the very low levels of bile salts remaining in the large intestine may have significant consequences for the temporal expression of virulence genes by pathogenic bacteria [46]. Bile salts also act as detergents due to their amphipathic properties, which makes them potent antimicrobial agents that disrupt cell membranes [47], damage DNA [48] and cause oxidative stress [49].

#### 2.2.2. Effect of Bile Salts on EHEC Survival/Physiological State

Adaptability to the harsh effects of bile acids is a critical component of survival for commensal bacteria and for GI pathogens. Enteric bacteria are known to alter protein production and increase mechanisms of resistance to survive the deleterious effects of bile [47], but whether this is a generalized stress response or a specific sensory phenomenon is unclear. *E. coli* in particular is adapted to the GIT and tolerates the presence of bile salts. Its mechanism of resistance is based on modified membrane structures that reduce bile permeability and an active removal of bile acids that have traversed the membranes into the cytoplasm [47]. Bile acids enter cells through the OmpF porin of the outer membrane, but are immediately expelled via efflux pumps [50].

Resistance to bile can be even greater in EHEC. In an inoculated bovine host model, EHEC O157:H7 was able to grow in 15% bile and pass from the gallbladder throughout the intestinal tract [51]. Other studies report the isolation of EHEC O157:H7 [52] from the gallbladder of calves, where the strain is highly persistent [53]. Moreover, bile salts stimulated the expression of genes associated with the acid-stress response of the bacteria [54].

#### 2.2.3. Effect of Bile Salts on EHEC Virulence

As previously mentioned for pH, bile salts are able to modulate various facets of EHEC virulence.

##### Motility

Bile treatment of EHEC O157:H7 affected mRNA levels for the entire flagella-chemotaxis regulon, resulting in a two- to four-fold increase in mRNA levels of genes associated with the flagella hook-basal body structure while causing a two-fold decrease in expression of “late” flagella genes associated with the flagella filament, stator motor, and chemotaxis [46]. However, the mechanisms for this remain unclear. In addition, the presence of bile salts reduced flagellin gene (*fliC*) transcription in O157:H7 in vitro [54]. Contradictory findings on the importance of flagella in EHEC pathogenesis have been reported, indicating that a flagellum is not playing the same role depending on the model tested.

##### Adhesion

Contradictory effects of bile concerning adhesion have been highlighted in different studies. While growth of EHEC O157:H7 strains in MacConkey agar containing 0.5% bile salts increased the expression of *lpf* by 2.7-fold [33], transcriptomic analysis showed that bile exposure reduced expression of the LEE pathogenicity island [46]. In accordance with the latter observations, Yin and colleagues showed that the presence of bile inhibited O157:H7 adherence to epithelial cells in vitro and repressed expression of LEE-associated genes [54].

##### Iron Uptake

Bile salts increased mRNA levels for 17 genes associated with iron scavenging and metabolism, and counteracted the inhibitory effect of the iron-chelating agent 2,2′-dipyridyl on the growth of EHEC O157:H7 [54]. These findings suggest that EHEC may use bile as an environmental signal to adapt to changing conditions associated with the iron-scarce environment of the small intestine [46].

##### Stx Phage Induction and Stx Production

Bile salts (sodium cholate and sodium deoxycholate) attenuated the excision of a subset of complete and truncated Stx bacteriophages from EHEC O157:H7 strains [55]. The *stx* expression is directly linked to the phage induction process, therefore if Stx phage is not induced, *stx* expression is not enhanced. The absence of Stx phage induction in the presence of bile salts is in accordance with Hamner and colleagues [46] who found no significant changes in *stx* gene expression in O157:H7 treated with 0.8% bile salts in non-inducing conditions [24]. All together, these findings suggest that bile salts are not influencing, or rather inhibiting, *stx* expression in EHEC.

### 2.3. Role of Low Oxygen Concentrations

#### 2.3.1. Oxygen Distribution in the Gut

The environment in the gut is characterized by varying levels of oxygen with concentrations decreasing from the stomach towards the distal colon. Several factors are likely to contribute to the development of this gradient including oxygen uptake by swallowing food, digestion-dependent blood flow and oxygenation of the intestinal mucosa, and oxygen consumption by the epithelium and gut microbiota [56,57]. In addition to longitudinal differences in oxygen levels along the GIT, a radial gradient with increasing oxygen concentrations from the gut lumen to the mucosal surface is evident which manifests itself in elevated levels of aerotolerant commensal bacteria associated with human intestinal mucosa compared to faeces [58].

#### 2.3.2. Oxygen and *E. coli* Fitness

As facultative anaerobes, commensal and pathogenic *E. coli* such as EHEC exhibit considerable flexibility in using aerobic and anaerobic respiration and fermentation to generate energy in the changing oxygen environment in the gut. In the presence of oxygen, *E. coli* can utilize two respiratory oxidases for aerobic respiration: whereas the low-affinity cytochrome *bo_3_* oxidase is active under aerobic conditions, the high affinity *bd* oxidase can still bind oxygen at nanomolar concentrations but generates less energy [59,60]. Under anaerobic conditions, *E. coli* can use a range of alternative terminal electron acceptors including nitrate, nitrite, trimethylamine *N*-oxide (TMAO), dimethyl sulfoxide (DMSO), or fumarate [61]. In the absence of alternative electron acceptors, *E. coli* can perform mixed acid fermentation generating a mixture of lactate, acetate, ethanol, succinate, formate, carbon dioxide, and hydrogen. However, this process is only used as a last resort as it is the least energetically efficient [62].

#### 2.3.3. Influence of Oxygen Levels on EHEC Virulence

##### Adhesion

Interestingly, EHEC O157:H7 grown in chemostat conditions demonstrated reduced growth and metabolic efficiency under anaerobic or oxygen-limited conditions compared to aerobic cultures [63]. EHEC grown under oxygen-limited conditions adhered significantly better to human epithelial HEp-2 cells compared to aerobic cultures suggesting the induction of adhesins in oxygen-restricted environments. While increased binding of anaerobic chemostat cultures of EHEC O157:H7 was independent of type 1 pili [63], low oxygen levels enhanced expression of sorbitol fermenting protein (Sfp) fimbriae and adherence to human intestinal Caco-2 and HCT-8 cells in sorbitol-fermenting EHEC O157:NM [64]. In addition, oxygen levels influence expression of the EHEC T3SS and/or secretion of effector proteins such as EspA. When EHEC was grown in LB medium, lower amounts of T3S proteins were detected under anaerobic versus aerobic conditions [65]. However, addition of the alternative terminal electron acceptors nitrate or TMAO, but not DMSO or fumarate, restored T3S to aerobic levels and resulted in increased EspA expression and A/E lesion formation on Caco-2 cells. Further studies demonstrated that anaerobic growth without electron acceptors resulted more frequently in a premature T3SS suggesting that activation of TMAO- or nitrate-specific respiration enhances maturation of a functional T3SS in the absence of oxygen [65]. Reduced expression of the EHEC T3SS at low oxygen levels was also observed in a recent study, where EHEC was grown in low glucose DMEM medium under anaerobic, microaerobic or aerobic conditions [66]. Importantly, oxygen availability affected how the transcription factors Cra, KdpE, and FusR modulated LEE gene expression: while KdpE and FusR repressed expression of the T3SS under anaerobic conditions, Cra activated LEE gene expression under aerobiosis [66]. This strategy would allow EHEC to fine-tune expression of the T3SS to the radial oxygen gradient in the intestine with minimal LEE gene expression (and energy expense) in the anaerobic gut lumen and targeted T3S and injection of effector proteins at the oxygenated epithelial surface. To simulate the microaerobic zone of oxygenation at the intestinal epithelium, a vertical diffusion chamber (VDC) system was used where a polarized monolayer of T84 colon carcinoma cells grown on a permeable membrane was inserted between two half chambers. While the apical side of the model epithelium was perfused with an anaerobic gas mixture and infected with EHEC, the basolateral side was maintained under aerobic conditions to simulate the oxygen supply by the bloodstream [67]. In this system, EHEC T3S and host cell adherence were enhanced under microaerobic versus aerobic conditions indicating that the presence of an epithelial oxygen gradient promotes EHEC A/E lesion formation in the human colon.

##### Stx Production

Initial studies on steady-state chemostat cultures demonstrated similar Stx levels during anaerobic and aerobic growth [63]. However, Stx production and release was enhanced under aerobic versus microaerobic conditions during EHEC infection of polarized T84 cells in the VDC system [68]. As previously mentioned, *stx* expression and release (particularly for Stx2) is largely governed by the induction of the phage lytic cycle which is triggered by DNA-damaging agents including reactive oxygen radicals [69,70]. As these are naturally produced during the sequential reduction of molecular oxygen in aerobic respiration [71], this might explain the increased production of Stx in the presence of oxygen. In addition to release of free toxin after bacterial lysis, Stx can be released within outer membrane vesicles (OMV) during bacterial growth [72,73]. Interestingly, anaerobic conditions promoted Stx2 release within OMV during EHEC culture in Casein Hydrolysate Yeast Extract (CAYE) broth while free toxin was prevalent in aerobic cultures [74]. The mode of Stx release could have an impact on the mechanism and efficiency of toxin penetration across the gut barrier, as human intestinal epithelium does not express the Stx receptor Gb_3_ [75] whereas OMVs can be internalised by host cells via receptor-dependent and -independent pathways [76]. Notably, VDC studies have shown enhanced Stx transport across polarized T84 cells during microaerobic versus aerobic EHEC infection [68], which could indicate enhanced toxin uptake via OMVs. Alternatively, low oxygen levels might promote the expression of bacterial factors facilitating toxin transport. As oxygen levels have a profound influence on EHEC growth, expression of the T3SS, Stx release and transport, it is important to reflect these conditions in experimental model systems when studying EHEC pathogenesis in the human gut.

### 2.4. Impact of Fluid Shear

#### 2.4.1. Fluid Shear in the Gut

Fluid shear can be defined as the distribution of frictional forces due to hydrodynamic flow generated by GI peristaltic activity against the surface of intestinal epithelial cells. In the human gut, there is a decreasing gradient of fluid shear stress from the mucosa to the gut lumen. Physiological levels of shear stress found in the intestinal epithelium during peristalsis may range between 35 and 0.02 dynes/cm^2^ [77]. Shear forces can approach 5 dynes/cm^2^ at the brush border surface and decrease to 2–3 dynes/cm^2^ between microvilli. Bacterial fimbriae are presumed to establish and maintain binding to target cells in the face of hydrodynamic shear forces generated by GI peristaltic activity.

#### 2.4.2. Effect of Fluid Shear on EHEC Virulence

Studies conducted in uropathogenic *E. coli* and *Pseudomonas* strains showed that shear stress inhibits pathogen adhesion, thereby serving as a non-specific host defence mechanism against bacterial colonization [78]. However, a novel mode of shear-enhanced, or so-called catch-bond, adhesion has been described in bacteria wherein the adhesive interactions become stronger rather than weaker under increasing shear conditions [79]. Type 1 fimbriae and FimH from *E. coli* are the main bacterial systems for which the structural aspects of catch-bond formation have been explored in detail, especially in uropathogenic *E. coli*. For EHEC O157:H7, Alsharif and colleagues have shown that initial attachment to host cells generated a mechanical cue, which was further enhanced by fluid shear level in the host GIT and was required to fully activate LEE-encoded virulence mechanisms [80] via the LEE-encoded global regulator (Ler). This study shows that, in addition to chemical signals, EHEC can sense and respond to mechanical cues to adapt to the physiology of the host and fine-tune virulence activation.

**Figure 2:**
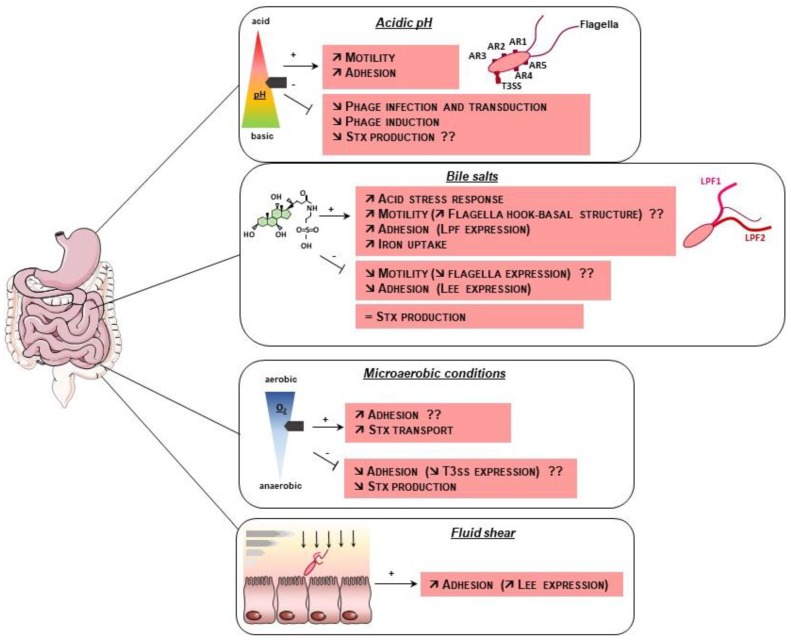
Influence of abiotic factors on EHEC virulence. EHEC has the ability to resist the stressful conditions encountered in the gut such as acidic pH and bile salts and utilizes various abiotic GI cues to modulate the expression of its virulence factors. Stx: Shiga-toxins, LPF: Long Polar Fimbriae, LEE: Locus of Enterocytes Effacement, T3SS: Type III secretion system, AR: Acid resistance, arrow with the sign + represents an activation process, bolded line with the sign − represents repression, ?? indicates contradictory results.

The effect of abiotic factors on EHEC virulence is summarized in Figure 2.

## 3. Effect of Biotic or Host Cell-Related Parameters

### 3.1. Interactions with the Gut Microbiota

#### 3.1.1. Importance of the Gut Microbiota

A dense population of microorganisms that has important physiological functions in the gut colonizes the human gut. One of these functions is to confer protection against invasion by pathogens. The molecular basis for this “colonization resistance” is multifactorial and involves several processes including competition for nutrients, stimulation of immune defenses and direct inhibition of pathogen expansion through production of inhibitory molecules and/or bacteriocins by commensal species [81,82]. Numerous studies have deciphered how bacterial pathogens circumvent colonization resistance in order to infect the GIT and induce disease. The diverse strategies include the use of specific nutrient sources different from those used by commensals, the alteration of gut normobiosis through induction of inflammation, and the direct killing of competitors via specific bacterial systems [83,84]. In the next section, we will focus on the interactions between the human gut microbiota and EHEC, as well as the strategies employed by the pathogen to overcome colonization resistance.

#### 3.1.2. Effect of the Gut Microbiota on EHEC Survival/Physiological State

One of the major aspects of colonization resistance is probably competition for nutrients between the gut microbiota and incoming pathogens. Indeed, Le Bihan and colleagues demonstrated that growth of EHEC O157:H7 strain EDL933 in filtrated caecal content of human gut microbiota-associated rats resulted in changes in expression of almost 20% of genes compared to growth in caecal contents of axenic rats [85]. Interestingly, most of these genes were associated with metabolic functions, indicating adaptation of EHEC to a limited nutrient environment by reprogramming and activation of metabolic pathways. Specifically, these studies have shown that EDL933 switches from a glycolytic to an anaplerotic metabolic profile characterized by the degradation of alternative carbon sources to supply intermediates of the Krebs cycle such as amino acids (tryptophan, phenylalanine, leucine), amino sugars (*N*-acetylneuraminic acid) or small aromatic compounds (3-hydroxyphenylpropionic acid; 3-hydroxycinnamic acid). While members of the Proteobacteria phylum, especially *Enterobacteriaceae*, represent a very small proportion of the total gut microbiota, they appear to have a key role in nutrient competition with most bacterial pathogens since they share the same nutritional niche. Using streptomycin-treated mice, several studies from the group of Cohen and Conway determined the sugar-defined niche of commensal and pathogenic strains of *E. coli* elegantly demonstrated that pre-colonization of animals with a combination of *E. coli* strains consuming the five carbohydrates preferentially used by EDL933, prevented gut colonization by the pathogen [86,87,88]. Similarly, germ-free mice colonized by certain commensal *E. coli* strains efficient in the utilization of proline were resistant to colonization by EHEC O157:H7, indicating another method for commensals to provide colonization resistance [89].

Apart from competition for nutrients, colonization resistance can be mediated by direct antagonism of commensals over pathogenic strains, and several strains of *E. coli*, *Lactobacillus reuteri*, *Clostridium butyricum* and *Bifidobacterium* sp. have been shown to inhibit EHEC growth [90,91,92,93]. Different types of molecules produced by gut microbes have been associated with growth inhibition and/or killing of EHEC including short-chain fatty acids (SCFA, see Section 3.3), lactic acid, hydroxypropionaldehyde and bacteriocins [92,94,95,96].

#### 3.1.3. Effect of the Gut Microbiota on EHEC Virulence

As previously mentioned, activities of microbial species from the gut microbiota influence the nature and concentrations of metabolites present in the gut ecosystem and may therefore affect virulence gene expression in pathogens. Numerous studies have investigated the impact of individual commensal species or, less frequently, of the entire gut microbiota on several aspects of EHEC infection. The next sections will highlight the most recent discoveries exemplifying how the gut microbiota can affect EHEC virulence.

##### Adhesion

Several studies have shown that *Lactobacillus* and *Bifidobacterium* species decrease EHEC adhesion to intestinal epithelial cells in vitro by different mechanisms including direct competition for binding sites at the enterocyte surface [97,98,99,100,101], production of molecules modulating expression of adhesins [102,103,104] and stimulation of mucus production, thereby preventing access to the epithelial surface [105,106]. Conversely, in vitro co-culture of EHEC O157:H7 and O103:H25 and the commensal strain *Bacteroides thetaiotaomicron* led to increased expression of all LEE operons [107,108], increased epithelial adhesion and formation of A/E lesions. The same effect was observed following co-incubation of EHEC O157:H7 and *Enterococcus faecalis*, a commensal strain belonging to the phylum Firmicutes [107]. In contrast, LEE gene expression in EDL933 was down regulated during EHEC growth in the filtrated caecal contents of human gut microbiota-associated rats [85].

At the molecular level, several studies pinpoint a role of quorum-sensing molecules in the control of LEE gene expression and adhesion to epithelial cells. Regulation of the LEE involves LuxS, which is required for the synthesis of autoinducers AI-2 and AI-3 [109,110,111]. The receptor for AI-3, QseC, also senses the eukaryotic hormone norepinephrine (NE) which is the main neurotransmitter of the sympathetic nervous system [112] (see Section 3.6). Moreover, NE can be transformed by the gut microbiota to 3,4-dihydroxymandelic acid (DHMA) which has been shown to be a chemoattractant for EHEC and to modulate expression of LEE genes as well [113]. DHMA is generated by the enzymes tyramine oxidase and aromatic aldehyde dehydrogenase, which are encoded by numerous members of the *Enterobacteriaceae* family. Another signaling molecule present at high concentrations in the GIT, indole, modulates EHEC virulence properties. Indeed, indole was found to exert divergent effects on EHEC chemotaxis, motility and enterocyte adhesion when compared to NE [114]. However, another study determined that the influence of indole on LEE expression depends on its concentration, with activating effects occurring at low concentrations (µM) and repressing effects at high concentrations (mM) [115]. Altogether, these results demonstrate that the influence of the gut microbiota (and consumed or generated metabolites) on EHEC virulence is dependent on the commensal strains, metabolite concentrations and experimental conditions used for each study. Since EHEC virulence gene expression in the intestine is governed by a myriad of environmental signals affected by the presence and/or activities of individual commensal species in the gut ecosystem, this reinforces the need to perform integrative investigations of EHEC behaviour within the gut, for example by using mathematical models to predict the outcomes of such a complex ecosystem [116,117].

##### Stx Production and Toxicity

Activities of the human gut microbiota also affect Stx production by EHEC. Indeed, in vitro growth of EDL933 in conditioned medium from a complex human gut microbiota, led to a lower synthesis of Stx2 when compared to growth in unconditioned medium [118]. This is mediated by inhibition of the lytic cycle via the SOS response regulator RecA. Interestingly, similar results were obtained when EHEC O157:H7 was cultured in medium conditioned by the predominant species of the Bacteroides phylum *B. thetaiotaomicron* only [108,118]. Furthermore, Cordonnier and colleagues demonstrated that reduced Stx2 production was dependent on internalization of vitamin B_12_ by *B. thetaiotaomicron* as a mutant deficient in an outer membrane receptor of vitamin B_12_ was unable to repress the Stx2 production. This work suggests that the concentration of vitamin B_12_ in the gut and by extension, activities of commensal bacterial species producing and/or consuming vitamin B_12_, may modulate EHEC Stx production and consequently the outcome of the infection. Other studies also demonstrated that the gut microbiota can affect the translocation of Stx across intestinal epithelium or increase the number of Gb3 receptor in murine tissues, thereby enhancing susceptibility to EHEC infection [119,120]. Whereas germ-free mice were susceptible to EHEC infection, mice mono-associated with *Bifidobacterium longum*, but not *B. adolescentis*, survived infection [119]. This species-specific effect relied on the presence of specific carbohydrate transporters in protective strains conferring the ability to consume specific carbohydrates. This resulted in the production of acetate, which was partly responsible for the limited translocation of Stx from the gut lumen into the blood observed in mice inoculated with protective *Bifidobacterium* strains. Research from another group has determined that mice fed with a high fiber diet were more susceptible to EHEC infection [120]. Gut microbiota of these mice contained reduced levels of native *Escherichia* species compared with mice receiving a low-fibre diet, which might facilitate EHEC colonization in the absence of competitors. The higher levels of butyrate in the GI contents of high fiber diet mice also induce the synthesis of Gb3 in both the gut and kidneys. This strongly suggests that susceptibility to EHEC infection also depends on diet and/or the capacity of the gut microbiota to produce SCFA (see Section 3.3). In conclusion, several in vitro and in vivo studies have demonstrated that the gut microbiota can modulate the production of Stx by EHEC and therefore can affect the outcome of an infection.

### 3.2. Interactions with Mucin Sugars

#### 3.2.1. The Mucus Layer in the Gut

The intestinal epithelium is covered by a complex layer of mucus mainly constituted of secreted oligomerized mucins which are large heavily O-glycosylated proteins [121]. The main monosaccharides found in human intestinal mucins are galactose, *N*-acetylgalactosamine (GalNAc), fucose and *N*-acetylneuraminic acid (Neu5Ac) [122]. Typically, these carbohydrates can be cleaved from mucins by members of the gut microbiota expressing specific enzymes (glycoside hydrolases, proteases and sulfatases) and become available as nutrients for themselves or surrounding bacteria. In the next section, we will discuss how the mucus, and particularly mucin carbohydrates, affects EHEC behaviour during the infectious process.

#### 3.2.2. Effect of Mucus on EHEC Fitness

While EHEC possesses all the genes required to utilize the five sugars present in mucins, it has been shown that only the consumption of fucose, galactose and GlcNAc is important for efficient colonization of the mouse gut by strain EDL933 [86]. As *E. coli* is not able to degrade complex polysaccharides, the action of mucinolytic commensals is needed for release and access of monosaccharides. This is the basis for the “restaurant” hypothesis established by the group of Conway and Cohen stipulating that *E. coli* colonizes the mucus layer within local bacterial communities including anaerobes which are able to break down polysaccharides [123]. Interestingly, EHEC, but not commensal *E. coli*, produces proteins that can facilitate the release of mucin sugars by mucinolytic commensals. Indeed, EHEC secretes the metalloprotease StcE which is able to cleave mucins and contributes to reduction of the mucus layer covering intestinal cells [124,125]. Another example is the presence of up to 10 prophage-located *nanS* genes (*nanS*-p) in the genome of O157:H7 strain EDL933 and O104:H4 strain LB226692 [126,127]. The *nanS*-p genes encode esterases involved in deacetylation of 5-*N*-acetyl-9-*O*-acetyl neuraminic acid (Neu5,9Ac_2_), another form of neuraminic acid present in mucus. This reaction leads to release of Neu5Ac which can then be internalized and metabolized by the pathogen. As additional clues of their contribution to EHEC pathogenesis, both *stcE* and *nanS*-p genes are co-expressed with LEE and *stx*_2_ genes, respectively [124,126]. Because intestinal mucins are heavily sulfated, a similar hypothesis can be attributed to a sulfatase-encoding gene co-regulated with the LEE genes in EHEC [128]. Altogether, these data strongly suggest that EHEC has developed tricks to efficiently access mucin-derived sugars and benefit from nutrient source to colonize the GIT.

#### 3.2.3. Effect of Mucus on EHEC Virulence

##### Adhesion

To adhere to the intestinal epithelium, EHEC must penetrate the mucus layer overlying the epithelium. Interestingly, a recent study revealed that adhesion of EHEC to mucin-producing epithelial cells was higher compared with mucin-deficient cell lines [125]. Similar observations have been reported for other intestinal pathogens such as enteropathogenic *E. coli* (EPEC) and *Salmonella enterica* [129,130]. No specific adhesin has been associated with EHEC binding to mucus-producing cells. However, the absence of flagella seems to be important for this interaction since a Δ*fliC* mutant adhered more efficiently than the wild-type strain [125], and expression of flagellar genes was repressed upon exposure to mucus [131]. Adhesion to mucus-producing cells was also markedly correlated with a decreased thickness of the mucus layer [125,132]. The reduction of mucin levels was dependent on the metalloprotease StcE since a Δ*stcE* mutant was not able to disrupt the mucus layer covering epithelial cells or human colonic biopsies [125]. Subtilase, a cytotoxin produced by some non-O157 EHEC strains, might also contribute to mucin depletion as observed following treatment of human colonic mucosa with an O113:H21 strain or with purified subtilase [133]. Alteration of the mucus layer by EHEC thereby promotes access to the epithelial surface and binding of the pathogen to enterocytes.

In addition to its ability to penetrate mucus, EHEC also regulates the synthesis of virulence factors involved in bacterial adhesion depending on the concentration of mucin sugars present in close proximity and by extent, on mucinolytic activities of commensals. Indeed, several sugar-sensing systems are involved in the control of both metabolic and LEE gene expression in EHEC. For example, the regulatory protein NagC, which represses GlcNAc and galactose catabolism in *E. coli*, also controls the expression of LEE genes via direct binding to the LEE1 promoter region [128]. Consequently, presence of GlcNAc or Neu5Ac in the medium inhibits EHEC adhesion to epithelial cells in vitro. This study also demonstrated that modification of GlcNAc concentrations in the GIT of mice, either by exogenous addition of the sugar or by release via activities of mucinolytic bacteria, affected the in vivo fitness of EHEC in a NagC-dependent manner. Another work identified a new two-component system in EHEC, FusKR, which is involved in the detection of external fucose as a signal to regulate expression of LEE genes [134]. Similar to GlcNAc or Neu5Ac, high concentrations of fucose repress production of T3SS and inhibit EHEC adhesion. Taken together, these studies suggest that regulation of LEE genes by mucin-derived sugars contributes to the relocation of the pathogen from the intestinal lumen to the epithelium surface. When crossing the outer mucus layer, EHEC senses high concentrations of free forms of sugars resulting from the activities of mucin-degrading commensal bacteria and, in response, represses the production of the T3SS which is not required at this stage. When the pathogen moves closer to the epithelium, sugars are mostly linked to mucins, and expression of LEE genes is activated in order to promote adhesion to epithelial cells. It is noteworthy that sugar catabolism and T3SS expression are not coordinated in the same way by GlcNAc and fucose. Whereas NagC activates *nag* and LEE genes, FusR represses both *fuc* and LEE genes [128,134]. The reasons for this difference remain unknown, and further studies are required while taking into account the complexity of the mucus structure in terms of composition, organization and thickness along the GIT.

##### Production and Toxicity of Stx

Among the Stx2 variants, Stx2d has a cytolytic activity that is increased 35- to 350-fold in the presence of intestinal mucus from several animals including humans and mice [135]. Originally, the *stx2d* gene has been discovered in O91 strains which are highly virulent in mice, but has now been detected in strains of other serotypes [136]. However, *stx2d* is mainly present in LEE-negative strains which are significantly associated with the development of bloody diarrhoea and HUS [137]. The group of O’Brien has published several studies deciphering how mucus increases the toxic activity of Stx2d on Gb3-expressing cells. They established that elastase present in mucus was responsible for the activation of Stx2d via cleavage of the toxin A subunit [138]. This event is determined not only by the presence of a sequence called “activatable tail” in the C-terminal part of the A2 subunit but also by the B pentamer associated with the A subunit [136,139]. Once activated, Stx2d shows a higher capacity to bind its receptor Gb3, leading to a higher toxicity towards target cells [140]. Taken together, these works highlight mucus as a signal sensed by EHEC in order to induce Stx2d production when reaching the gut epithelium.

### 3.3. Effect of gut Microbiota Metabolites

#### 3.3.1. Short Chain Fatty Acids (SCFAs)

As previously mentioned, the human GIT is a complex microenvironment containing a variety of substances, many of which are metabolic products from resident bacteria. These substances include a variety of SCFAs [141], which are the end products of fermentation of dietary fibres. Most bacterial activity occurs in the proximal colon, where substrate availability is highest.

SCFAs consist of one to six carbon units, of which acetate (C2), propionate (C3), and butyrate (C4) are the most abundant (≥95%) [142]. The Bacteroidetes phylum mainly produces acetate and propionate, whereas the Firmicutes phylum releases butyrate as primary metabolic end products. SCFA compositions and concentrations vary according to the segment of the intestine, from the ileum to the colon, as well as the type and amount of fibres in the diet [142]. The time after ingestion also has an effect, as SCFAs are absorbed rapidly.

#### 3.3.2. Effect of SCFAs on EHEC Survival/Physiological State

In general, SCFAs seem to enhance EHEC colonization by providing a substrate that can be fermented by EHEC in the large intestine [141]. Zumbrun and colleagues showed that mice fed a high fibre diet (with high levels of butyrate) exhibited a 10- to 100-fold increase in EHEC O157:H7 colonization [120]. However, EHEC colonization was affected by SCFA composition and concentrations [143], as well as by oxygen levels in the intestinal tract in which the effects were evaluated.

#### 3.3.3. Effect of SCFAs on EHEC Virulence

##### Motility

Under aerobic conditions, the presence of SCFAs activated the flagellar gene regulatory cascade and enhanced motility in EHEC [119]. The activation of flagellar genes was achieved via a response of two of three regulatory steps for flagellar genes. The class I pathway involves *flh*DC regulatory genes via the leucine-responsive regulatory protein (Lrp) and is activated by butyrate. Moreover, butyrate, acetate and propionate can activate class II regulation of flagellar genes by acting on genes downstream of *flh*DC and independently of *flh*DC activation [119]. In addition to flagellar synthesis, Lrp also regulates LEE virulence gene expression [144], and Tobe and colleagues reported induction of flagellar and LEE genes by SCFAs in the intestine. The simultaneous activation of motility and adhesion appears contradictory. Whereas most adherent bacteria harbour flagella at the initial stage after growth in the presence of SCFAs, flagellar expression is switched off during microcolony formation [144]. This might be due to the inhibitory effect of the LEE, which is also activated by butyrate.

SCFA levels are lowest in the small intestine and increase in the large intestine. It has been hypothesized that EHEC activates flagellar production and motility when encountering rising concentrations of SCFAs in the small intestine, whereas, in the colon, elevated SCFA levels provide sufficient butyrate to induce LEE gene expression [144] and repress flagellar synthesis. Lackraj and colleagues supported this model by showing a significant induction or repression of flagellin expression and motility in EHEC exposed to mixtures of SCFAs representative of the small intestine and colon, respectively [145]. This agrees with the large intestine being considered the preferred EHEC colonization niche, and once reached, the transit mediated by motility factors is interrupted by a switch from the swimming motile phenotype to host adhesion.

Other studies reported that acetate, propionate, or combinations of both, reduced EHEC O157:H7 motility under anaerobic conditions [146]. Interestingly, combinations of lactate and acetate had the same effect, but resulted in an increase in motility under aerobic conditions [89]. This suggests that not only the presence of SFCAs but a combination of intestinal environmental factors, play a role in the regulation of EHEC motility.

##### Adhesion

Simulation of SCFA concentrations found in the human colon induced expression of the gene *iha*, which encodes an adherence-conferring outer membrane protein of pathogenic *E. coli* [141]. However, these studies were conducted under shaking aerobic conditions. When the assay was carried out in a 5% CO_2_ atmosphere [145], closer to the oxygen concentration in the intestinal lumen, no significant changes or down-regulation of *iha* expression was observed.

As mentioned in Section 3.1.3, butyrate can up-regulate LEE gene expression in EHEC, favouring adhesion by enhancing the expression of the intimate attachment T3SS and effector proteins [104]. In the proposed model, the concentration of SCFAs produced by the gut microbiota gradually increases toward the large intestine, where butyrate levels are highest, and cause the corresponding increase in expression of LEE and associated genes prepares EHEC for adherence [104,144]. Once EHEC reach the surfaces of epithelial cells and establish initial attachment, the production of flagella is turned off as explained before, because they are no longer needed in the infection process. In fact, flagella at this stage would be an impediment to successful infection, since flagellin is a strong agent in inducing a host pro-inflammatory response.

##### Iron Uptake

As mentioned above, SCFAs stimulate *iha* gene expression in EHEC [141]. Interestingly, Iha from UPEC strain UCB34 functions as a catecholate siderophore receptor when cloned in *E. coli* K-12 [147]. Additionally, *iha* expression is repressed in the presence of iron via the ferric-uptake regulator protein Fur [148]. This data suggests that SCFAs could enhance iron uptake by EHEC, but this needs further investigations.

##### Production and Toxicity of Stx

While the effect of SCFAs on Stx phage infection and induction has not been investigated, propionate or acetate produced by the anaerobic intestinal bacterium *B. thetaiotaomicron* did not significantly affect Stx production [149]. Other studies showed that acetate produced by *Bifidobacterium* protected mice against EHEC O157:H7 infection by inhibiting Stx translocation from the gut lumen into the bloodstream [119]. Despite a lack of evidence of a direct effect of SCFAs on Stx production, high concentrations of butyrate as a consequence of a fibre-rich diet increase EHEC colonization (see above), which in turn enhances the amount of Stx produced [120].

Butyrate has a profound effect on cell morphology and function, acting as a primary energy source for colonocytes, and increasing Gb3 expression in endothelial cells. Butyrate and other SCFAs produced in the large bowel are rapidly absorbed, transported to the liver via the portal vein, and then circulated through the body to the kidneys [150]. The kidney naturally expresses high levels of Gb3, which can be further increased by butyrate resulting in enhanced Stx sensitivity and cytotoxic damage [120,150]. High levels of butyrate could therefore pose a risk by increasing the susceptibility to Stx observed in both the intestine and kidney.

### 3.4. Role of Nitric Oxide

#### 3.4.1. Nitric Oxide in the Gut

Nitric oxide (NO) is a simple gaseous molecule that participates in a vast number of physiological processes, including vasodilatation and nerve transmission, in almost all organs of the human body. Focusing on gut physiology, NO is critical for GI motility [151] and plays a key role in the immune response to bacterial, viral, fungal and parasitic infections [152,153].

#### 3.4.2. NO Detoxification

NO is produced from l-arginine by NO synthase enzymes (NOS) expressed in many types of cells including immune cells as well as epithelial and endothelial cells. As a highly reactive free radical, NO can react with a large spectrum of molecules such as inorganic elements, DNA, proteins and lipids, thereby resulting in strong antimicrobial activity [154]. *Enterobacteriaceae* and in particular *E. coli* possess several resistance mechanisms against exogenous NO released by the mammalian host. Four NO detoxification pathways have been characterized in *E. coli* so far. These are mediated by the flavohaemoglobin HmpA [155], the flavorubredoxin NorV [156], the hybrid cluster protein Hcp–Hcr, and the nitrite reductases NrfA and NirB [157,158]. All enzymes use NO or NO derivatives as a substrate, thus allowing *E. coli* to resist NO cytotoxic effects. In EHEC, the contribution of the *norVW* operon to NO resistance has been investigated in more detail.

Initially, it has been observed that O157:H7 strains associated with a higher risk for the development of HUS possess an intact *norV* gene while a gene having a 204 bp deletion was detected in less virulent O157 strains, thus yielding an inactive form of the NO reductase NorV [159,160]. However, a more recent epidemiological study comparing O157:H7 strains isolated from HUS patients or asymptomatic carriers does not confirm a positive correlation between the presence of an intact *norV* gene and the disease severity [161]. Nonetheless, Shimizu and colleagues demonstrated that O157:H7 strains carrying an intact *norV* gene were protected from NO-mediated growth inhibition under anaerobic conditions [162]. Moreover, these strains exhibited higher survival within macrophages compared to *norVs*-type EHEC strains having the truncated form of *norV* suggesting that the flavorubredoxin has a role in EHEC pathogenesis.

#### 3.4.3. Effect of NO on EHEC Virulence

##### Adhesion

NO inhibits EHEC adherence to epithelial cells cultured in vitro. In particular, it has been shown that the NO-sensing regulatory protein (NsrR) is a direct activator of LEE1, LEE4 and LEE5 operons and that the presence of NO decreases the expression of the T3SS [163]. Such anti-adhesive properties of NO have been confirmed in vivo as EHEC-infected mice treated with an NO synthase inhibitor demonstrated enhanced EHEC colonic mucosal colonization compared with untreated mice (G. Jubelin, unpublished results). Notably, NO also affects other adhesion-associated processes such as enhanced dispersal of O157:H7 biofilms on abiotic surfaces [164].

##### Production of Stx

Vareille and colleagues demonstrated that sub-inhibitory concentrations of NO derived from chemical or cellular sources decreased Stx2 synthesis via NsrR [165]. In contrast, other authors reported that NO enhanced the production of Stx1 and Stx2 during EHEC O157 growth under anaerobic conditions [166].

Other EHEC virulence determinants are probably also affected by NO since they are controlled by regulatory proteins with iron-sulfur clusters that are well known to react very efficiently with NO [167,168]. If NO clearly interferes with EHEC virulence as previously described, the pathogen is able, in turn, to modulate the level of NO produced by epithelial cells. Vareille and colleagues showed that EHEC, in contrast to commensal or K12 strains, down-regulates NO production in IFN-γ induced human enterocytes cultured in vitro, thus limiting the detrimental impact of NO on virulence [169]. It is tempting to speculate that the level of NO produced as a biological output from the interplay between EHEC and the host might play a key role to determine the outcome of the infection.

### 3.5. Role of Ethanolamine

#### 3.5.1. Metabolism of Ethanolamine in the Gut

The GIT of mammals provides a rich and natural source of ethanolamine [170]. Indeed, ethanolamine arises from the digestion of phosphatidylethanolamine by bacterial phosphodiesterases [171]. After phosphatidylcholine, phosphatidylethanolamine is the most abundant phospholipid in cell membranes of animals and plants [172,173]. The utilisation of ethanolamine was investigated very early in *E. coli* where it was shown to involve a key coenzyme vitamin B_12_-dependent ethanolamine ammonia-lyase [174,175]. Actually, the metabolic pathway for degradation and utilisation of ethanolamine is encoded by an operon of 17 genes called *eut* (ethanolamine utilisation) where the ethanolamine ammonia-lyase is encoded by *eutBC* [176]. The ethanolamine ammonia-lyase is the first enzyme of this catabolic pathway which converts ethanolamine to acetaldehyde in the presence of cofactor vitamin B_12_ (cobalamin). From there, acetaldehyde can be converted to (i) ethanol by the alcohol dehydrogenase EutG, or (ii) acetyl-CoA by the acetyl dehydrogenase EutE before joining the tricarboxylic cycle (TCA) [176]. Alternatively, the phosphotransacetylase EutD can convert acetyl-CoA to acetyl-phosphate and ultimately to acetate by the acetate kinases EutP and EutQ [177]. In addition to regulators, several other proteins encoded by the *eut* operon (EutK, L, M, N, and S) participate in the formation of a bacterial micro-compartment dedicated to the catabolism of the ethanolamine, the ethanolamine metabolosome [178].

#### 3.5.2. Effect of Ethanolamine on EHEC Fitness

While ethanolamine is considered a valuable carbon and nitrogen source for bacteria [179,180], EHEC O157:H7 can only use ethanolamine as a nitrogen source [181]. While ethanolamine was poorly used by the endogenous intestinal microbiota, ethanolamine utilisation conferred a competitive advantage to EHEC O157:H7 in bovine intestinal contents [181]. Nonetheless, genes encoding components of ethanolamine metabolism have been identified in several bacterial genomes, including numerous species of the family *Enterobacteriaceae*, including α-proteobacteria, β-proteobacteria, δ-proteobacteria and γ-proteobacteria, and also in some other phyla, such as Firmicutes, Actinobacteria, Acidobacteria, Fusobacteria or Chlorophlexi [182]. This suggests that some kind of competition could occur between EHEC and other species of the gut ecosystem, which would require further investigations to comprehend the metabolic flux of ethanolamine in the different biotopes and communities encountered along the GIT.

While EHEC O157:H7 specifically binds to phosphatidylethanolamine, it cannot associate with the two other major phospholipids present in the epithelial cell membrane, phosphatidylserine or phosphatidylcholine [183,184]. As such, the specific recognition of the ethanolamine residue present in the hydrophilic head of some phospholipids could contribute to the colonization of the GIT by EHEC.

#### 3.5.3. Effect of Ethanolamine on EHEC Virulence

In EHEC O157:H7, ethanolamine was further shown to regulate the expression of virulence factors [185]. In particular, it acts as a signalling molecule and activates the expression of the LEE and *stx*. This regulation of virulence gene expression occurs independently of ethanolamine metabolism.

### 3.6. Interactions with Host Hormones

#### 3.6.1. Microbial Endocrinology

Microbial endocrinology is an emerging field aiming at comprehending the influence of hormones on the pathogenesis of infectious disease [186,187]. In the GIT, intestinal epithelial cells produce neurochemicals such as the catecholamine dopamine [188]. Catecholamines derive from phenylalanine or tyrosine and are formed around a benzene ring with two adjacent hydroxyl groups and an opposing amine side chain [186]. From the metabolic intermediate dopamine, different hormones can be formed such as adrenaline and noradrenaline (also called epinephrine and norepinephrine, respectively). The effect of these two hormones on EHEC survival and virulence is described below (Section 3.6.2).

#### 3.6.2. Effect of Host Hormones on EHEC Survival and Virulence

##### Survival

It has been shown that noradrenaline, adrenaline and dopamine can increase the growth of several commensal *E. coli* strains, as well as EHEC O157:H7 [189,190,191,192]. While originally observed in vitro in lab cultures, this effect was further confirmed in vivo using a murine model [193].

##### Adhesion

In vitro studies demonstrated that noradrenaline and adrenaline activate the T3SS in EHEC O157:H7 [110]. This was confirmed in a bovine ligated ileal loop model of infection where noradrenaline augmented EHEC O157:H7 adherence to intestinal mucosa and associated intestinal inflammation dependent on the ability of the pathogen to form A/E lesions [194]. In contrast, using Ussing chambers, noradrenaline promoted caecal adherence of EHEC O157:H7 strains defective in intimate mucosal attachment in mice [195]. In addition, significant enhancement of luminal attachment of EHEC O157:H7 to the colon through interactions with mucosal α-adrenoceptors and melanocortin receptors was also observed upon contraluminal application of noradrenaline [195,196]. While other intestinal hormones such as gastrin, galanin, and secretin did not influence LEE expression, their implication in the regulation of other bacterial physiological functions cannot be completely excluded. Lastly, in enterotoxigenic *E. coli* (ETEC) and commensal *E. coli* strains, noradrenaline increased the expression of pili involved in bacterial adhesion and biofilm formation to biotic and abiotic surfaces [190,197].

##### Iron Uptake and Stx Production

In addition to adhesion, noradrenaline induces the expression of the siderophore enterobactin as well as iron uptake [198,199,200], and also increases the production of Stx in EHEC O157:H7 [190]. Interestingly, expression of several major EHEC virulence factors including Stx, the T3SS and flagella is activated by a bacterial cell-to-cell signalling mechanism involving the autoinducer AI-2 synthesised by LuxS [109,201]. Previous studies have shown that noradrenaline and adrenaline can interfere with the LuxS/AI-2 system suggesting a potential cross-communication between bacterial and host cell signalling systems [110]. In addition to tyrosine-derived catecholamines, host signalling is mediated by polypeptide and steroid hormones, and much remains to be learned about the potential implications of these different hormones on the regulation of EHEC virulence and physiopathology.

**Figure 3:**
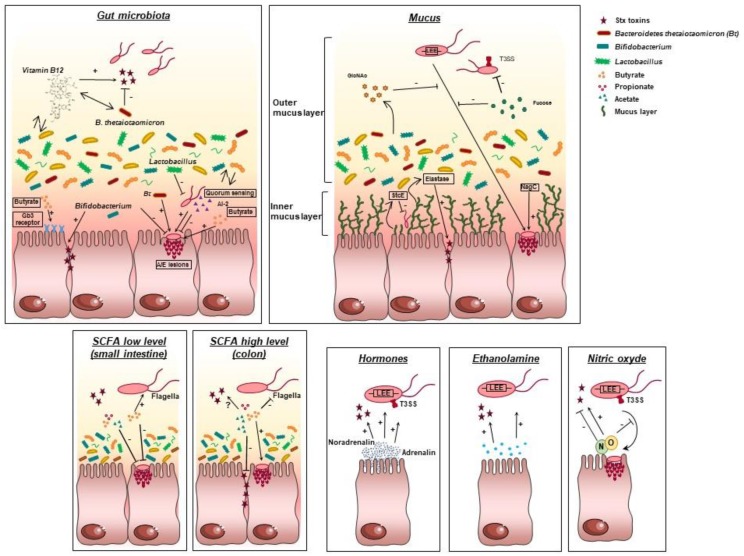
Influence of biotic factors on EHEC virulence. EHEC have evolved several strategies to modulate the expression of their virulence factors in response to biotic factors from the gut microenvironments. Each panel represents the interactions of EHEC with one biotic factor. Bt: *Bacteroidetes thetaiotaomicron*, LEE: Locus of Enterocytes Effacement, NagC: N-acetylglucosamine sensor, SCFA: Short Chain Fatty Acids, Gb3: globotriaosylceramide, T3SS: Type III secretion system, StcE: metalloprotease, arrow with the sign + represents an activation process, bolded line with the sign − represents repression, ? indicates contradictory results.

The effect of biotic factors on EHEC virulence is summarized in Figure 3.

## 4. Application of Dynamic Human Gut Models to Address Knowledge Gaps in EHEC Physiopathology

### 4.1. Limitation of Current Approaches

To be fully pathogenic, bacteria must not only survive in the human GIT but also coordinate expression of virulence determinants in response to localized gut microenvironments. As reviewed in this article, an increasing number of in vitro and in vivo studies have shown that EHEC can adapt to various environments found in the gut by detecting cues from the host or its resident microbiota. These cues are extremely diverse in nature, including biological, chemical, hormonal and mechanical signals, and can influence both pathogen survival and virulence. As illustrated in this review, most EHEC virulence genes are highly controlled by one or several of these environmental signals, and we are just starting to understand how the pathogen uses these cues in order to temporally and spatially coordinate/fine-tune virulence gene expression in the gut.

Despite obvious scientific progress in this area, the data obtained until now still show gaps and even some inconsistencies. Most of the models used are undoubtedly very useful to dissect specific mechanisms but do not reproduce human GI physiology. There are clear anatomical, biochemical, metabolic, and microbial differences between the GIT of mice and that of humans, and most in vitro studies performed have used oversimplified models reproducing only one digestive parameter at a time (e.g., acid pH, bile or SCFA). Therefore, integration and sequential delivery of GI signals is needed to model the dynamics and complexity of the human gut more closely. In particular, simulation of gastric pH drop, dynamics of digestive compartment emptying and real GI transit time, sequential delivery of digestive secretions and bile, and reproduction of a highly complex gut microbiota from human origin, are some of the key parameters required to strengthen the conclusion of the studies described in this review (see Section 2.1 for gastric pH, Section 2.2 for bile salts, Section 3.1 for gut microbiota and Section 3.3 for SCFA).

### 4.2. Main Dynamic Gut Models

As EHEC volunteer studies are ethically prohibited, an alternative to mimic human digestive physiology is the use of bio-regionalised and dynamic artificial digestive systems which reproduce the successive environmental niches of the human gut. Despite the large number of gut models available, only a small fraction combines dynamism and multi-compartmentalisation. Among them, the TIM (TNO gastrointestinal Model) [202,203] and the SHIME (Simulator of the Human Intestinal Microbial Ecosystem) [204,205] are particularly relevant as they benefit from more than 20 years of use and validation by in vitro/in vivo correlation studies, especially in the field of microbiology (but also for nutritional or toxicological applications). The TIM is currently the most complete simulator of the human upper GIT and consists of four compartments representing the stomach and the three parts of the small intestine (duodenum, jejunum and ileum). The TIM system addresses major physicochemical parameters of digestion including body temperature, temporal and longitudinal changes in gastric and intestinal pH, dynamism of chyme transit and mixing, sequential delivery of gastric (lipase, pepsin) and intestinal (pancreatic juice and bile) secretions and passive absorption of small molecules and water. Similarly, the SHIME is a multi-step fermentation model with five bioreactors that includes all compartments from the stomach to the colon (stomach, small intestine, ascending, transverse and descending colon). The bioreactors are maintained anaerobically by gassing with nitrogen and inoculated with fresh faecal samples from human volunteers. After a suitable adaptation period of the microbiota to the in vitro environmental conditions, the bioreactors run under continuously controlled conditions including pH, temperature, residence time, pressure, redox potential, agitation and nutrient availability. By incorporating mucin-covered agar beads in the colonic compartments, the SHIME model has been recently optimized (M-SHIME) to support establishment of the mucosal microbiota which fundamentally differs from the luminal community of resident bacteria [206]. TIM and SHIME emerge as a relevant alternative to in vivo assays, especially in relation with the study of foodborne pathogens such as EHEC.

### 4.3. Potential of Dynamic Gut Models in EHEC Studies: First Proofs

Dynamic gut models could be advantageously used to assess the effect of GI passage on EHEC survival and virulence in a temporal-spatial fashion (Table 1 and Table 2).

#### 4.3.1. Survival

Up to now, there is no clear data on how EHEC can survive after ingestion throughout the human GIT and which parameters influence this survival. Recent studies in the TIM and ARCOL (a single-step dynamic model of the human colon) models have allowed to provide such information for EHEC O157:H7. In the TIM, the survival of O157:H7 strain EDL933 was reduced in the stomach and duodenum, while bacterial growth was observed at the end of digestion in the jejunum and ileum [207]. This growth renewal in the distal parts of the small intestine was probably linked to the presence of less stringent conditions, such as neutral pH and lower concentrations of bile salts due to their reabsorption in the distal parts of the small intestine. Additional studies performed in ARCOL indicated that EHEC was not able to persist in colonic compartments [208,209] suggesting that EHEC colonization of the colon during human infection may be rather linked to growth renewal of the pathogen in the distal small intestine than its ability to maintain growth in the colon. Further studies in the TIM model have demonstrated enhanced survival of EHEC O26:11 in the human simulated upper GIT after ingestion via raw milk cheese compared with EHEC O157:H7 [210]. In addition, increased numbers of EHEC O157:H7 were recovered from the distal small intestine in vitro under infant digestive conditions compared with adult ones [211].

#### 4.3.2. Virulence

Initial results have also been obtained in relation to temporal/spatial regulation of EHEC O157:H7 virulence genes in the in vitro gut. Under adult conditions, both *stx1* and *stx2* were highly expressed in the ileal effluents of the TIM model and in the colonic compartment of ARCOL (until 12h post-infection), but not in the gastric compartment, in accordance with the main site of EHEC colonization in the terminal ileum and colon [208,211]. Interestingly, *stx1* and *stx2* expression in O157:H7 was significantly enhanced under infant digestive conditions versus adult ones in the TIM model, resulting in a higher amount of Stx produced in the ileal effluents [211]. These results combined with those previously mentioned (Section 4.3 paragraph survival) for survival indicate that age-related differences in digestive physicochemical parameters may partly explain the higher susceptibility of young children to EHEC infection and HUS. Notably, expression of the adhesins intimin gene *eae* and *lpf* was also activated in the TIM model during transit through the stomach and small intestine, despite the absence of epithelial cells [211,212]. In contrast, *eae* but not *lpf* was overexpressed in the colonic ARCOL model until 9h post-infection, suggesting that further studies are required to decipher the complex regulation of the EHEC adhesion process along the gut.

#### 4.3.3. Interaction with the Gut Microbiota

Up to date, all studies investigating the interactions between EHEC and the gut microbiota have been carried out using isolated commensal strains or complex microbiota from mice but not from human origin (only media conditioned by human microbiota). The only study investigating the interactions of EHEC with a complex human gut microbiota under physiologically relevant colonic conditions (e.g., pH, residence time, supply of nutrients found in human diet) was conducted in the ARCOL model using faeces from three different healthy volunteers [208]. While EHEC O157:H7 affected the composition of the colonic microbiota in an individual dependent manner (as assessed by qPCR analysis of the main bacterial communities), no changes in SCFA production were detected. Additional experiments are needed to conclude if differential gut microbiota composition may contribute to host resistance or susceptibility to EHEC infections. Then, differences in diet (as previously shown by Zumbrun and colleagues [120], see Section 3.1.3) and antibiotic regimens, which cause shifts in the composition of the GI microbiota may certainly influence the outcome of the disease.

### 4.4. Potential of Dynamic Models in EHEC Studies: Future Outlooks

As the parameters of dynamic gut models (such as TIM or SHIME) can be adjusted in terms of food-matrix and target group (age-range), we can consider unlimited applications of these systems to assess how serotype/strain, food vehicle, level of contamination (supra-physiological dose or real low infectious dose) or age (infant, adult, elderly) can affect EHEC survival or virulence under human GI conditions. EHEC virulence could be investigated through strategies targeting specific genes as previously described in the TIM model [211,212] or by global approaches such as microarray or RNA-sequencing. Moreover, we could also use the potential of these systems to assess whether Stx phages can improve the ecological fitness of EHEC. Phage induction can be monitored using reporter genes or quantification of Stx phage particles, and Stx transduction to other enterobacteria from the gut microbiota can be followed by using recombinant phages. The gut models also provide an efficient platform for evaluating anti-infectious strategies against EHEC infection, such as Stx-neutralizing antibodies/compounds, phage therapy, probiotics, prebiotics or manipulation of diet [207,208]. Lastly, they constitute a relevant complement to animal studies to decipher the role of the human gut microbiota on EHEC pathogenesis. The results obtained by Thévenot and colleagues [208] should be strengthened by additional experiments using a larger number of volunteers, from different age groups (especially infants), and also HUS patients. Given the effect of intestinal mucus on EHEC fitness and virulence (see Section 3.2), such studies would benefit from the M-SHIME model, which not only integrates the luminal microbiota but also reproduces the mucosal microenvironment. The composition of the luminal and mucosal microbiota can be analyzed by 16S rRNA sequencing and metagenomics, and specific use of nutrients by EHEC (e.g., derived from host mucins) can be determined by stable isotope probing or chemical imaging.

Although dynamic multi-compartmental systems are highly sophisticated, they do not reproduce the immune, nervous or hormonal control of digestion. Therefore, the combination of gut models and cell cultures (e.g., intestinal or immune cells) represents a common approach for integrating in vitro host responses [215,216]. One such model is the Host-Microbiota Interaction (HMI) module [216] which closely mimics the interactions and shear forces occurring at the interface of the mucus and epithelial surface. This HMI module is maintained under micro-aerobic conditions and can be used in combination with commercially available dynamic gut simulators, such as the SHIME system. Given the importance of oxygenation levels and fluid shear forces (see Section 2.3 and Section 2.4) on EHEC virulence, the HMI can also contribute to the mechanistic understanding of host, EHEC and microbiota interactions under physiologically relevant GI conditions.

Collectively, the data provided by the dynamic gut models, eventually combined with host cell components, will be essential not only for a full understanding of EHEC pathogenesis but also for setting regulatory standards in the food industry. They will also help identify host factors that influence the competence of the pathogen and help to define biomarkers for host susceptibility.

## Figures and Tables

**Table 1:** Description of the main dynamic in vitro models of the human gut used to address EHEC physiopathology.

Models	TIM-1	ARCOL	SHIME
**Main features**	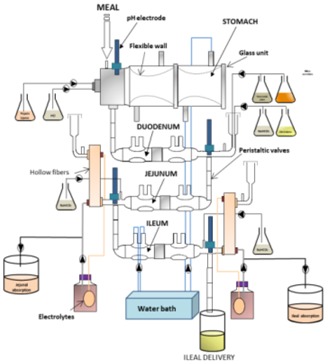 [202,203]	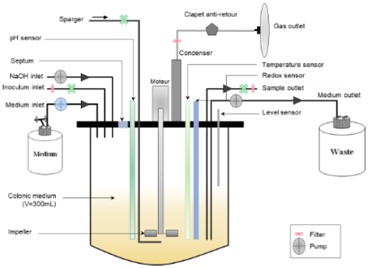 [202,208,209]	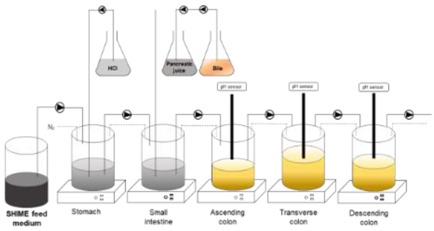 [204,205]
**Applications of dynamic models in EHEC studies**	• Survival of EHEC O157:H7 [207] • Survival of EHEC O26:H11 [210] • Regulation of *stx*, *lpf* and *eae* genes in the upper GIT [211,212] • Impact of age (infant versus adult) on EHEC survival and virulence [211] • Effect of probiotic yeasts on O157:H7 survival [207]	• Survival of EHEC O157:H7 [208,209] • Regulation of *stx* and *eae* genes [208] • Impact of O157:H7 on gut microbiota composition (qPCR) and activity (AGCC) [208,209] • Donor effect (3 different donors) [208] • Effect of probiotic yeasts on O157:H7 survival and virulence [208,209]	
**Perspectives**	• Stx phages and EHEC fitness • Effect of anti-infectious agents on EHEC survival and virulence (e.g., probiotics) • Impact of food matrix • Impact of strain/serotype and dose • Integrate the mucosal environnment Integrate age-related parameters (elderly people) • Integrate the host response => Combination with epithelial cells [213]	• Increase the cohort of volunteers • Use global approach for analyses of gut microbiota composition and functionality, and for analysis of EHEC virulence • Effect of anti-infectious agents on EHEC survival and virulence (e.g., pro- and prebiotics) • Integrate age-related parameters (infant) • Integrate the mucosal environnment • Reproduce pathological conditions (HUS)	• Investigate the interactions between EHEC and luminal/mucosal microbiota => M-SHIME [206] • Integrate the host response and evaluate the effect of microaerobiosis and fluid shear => HMI [214] • Integrate age-related parameters (infant, elderly) • Reproduce pathological conditions (HUS) • Effect of anti-infectious agents on EHEC survival and virulence (e.g., probiotics, prebiotics…)

**Table 2:** Roles played by in vitro dynamic models to address important questions related to EHEC pathogenesis in the human gut.

Main Questions That Remain to Be Addressed	TIM-1	SHIME
What is the impact of food vehicles on EHEC survival and virulence in the human GIT?	X	
How EHEC phages behave in the human GIT?	X	
Are there some strains or serotypes that better survive than others in the human GIT or produce more toxins?	X	X
Can inter-individual differences in physicochemical or microbial parameters of the human GIT influence EHEC survival or virulence?	X	X
Does EHEC infection affect human gut microbiota and may lead to dysbiosis?		X
How the oxygen level may influence EHEC virulence in presence of a complex gut microbiota?		X
